# PTEN in Lung Cancer: Dealing with the Problem, Building on New Knowledge and Turning the Game Around

**DOI:** 10.3390/cancers11081141

**Published:** 2019-08-09

**Authors:** Anastasios Gkountakos, Giulia Sartori, Italia Falcone, Geny Piro, Ludovica Ciuffreda, Carmine Carbone, Giampaolo Tortora, Aldo Scarpa, Emilio Bria, Michele Milella, Rafael Rosell, Vincenzo Corbo, Sara Pilotto

**Affiliations:** 1 Department of Diagnostics and Public Health, Section of Pathology, University of Verona, 37134 Verona, Italy; 2 Medical Oncology, Azienda Ospedaliera Universitaria Integrata, University of Verona, 37134 Verona, Italy; 3 Medical Oncology 1, IRCCS-Regina Elena National Cancer Institute, 00144 Rome, Italy; 4 Comprehensive Cancer Center, Fondazione Policlinico Universitario Agostino Gemelli IRCCS, 00168 Rome, Italy; 5 Medical Oncology, Università Cattolica Del Sacro Cuore, 00168 Rome, Italy; 6 SAFU Laboratory, Department of Research, Advanced Diagnostics, and Technological Innovation, IRCCS-Regina Elena National Cancer Institute, 00144 Rome, Italy; 7 Center for Applied Research on Cancer (ARC-NET), University of Verona, 37134 Verona, Italy; 8 Germans Trias i Pujol, Health Sciences Institute and Hospital, Campus Can Ruti, 08916 Badalona, Spain

**Keywords:** PTEN, lung cancer, NSCLC, genetic, epigenetic, treatment resistance, survival

## Abstract

Lung cancer is the most common malignancy and cause of cancer deaths worldwide, owing to the dismal prognosis for most affected patients. Phosphatase and tensin homolog deleted in chromosome 10 (PTEN) acts as a powerful tumor suppressor gene and even partial reduction of its levels increases cancer susceptibility. While the most validated anti-oncogenic duty of PTEN is the negative regulation of the PI3K/mTOR/Akt oncogenic signaling pathway, further tumor suppressor functions, such as chromosomal integrity and DNA repair have been reported. PTEN protein loss is a frequent event in lung cancer, but genetic alterations are not equally detected. It has been demonstrated that its expression is regulated at multiple genetic and epigenetic levels and deeper delineation of these mechanisms might provide fertile ground for upgrading lung cancer therapeutics. Today, PTEN expression is usually determined by immunohistochemistry and low protein levels have been associated with decreased survival in lung cancer. Moreover, available data involve *PTEN* mutations and loss of activity with resistance to targeted treatments and immunotherapy. This review discusses the current knowledge about PTEN status in lung cancer, highlighting the prevalence of its alterations in the disease, the regulatory mechanisms and the implications of PTEN on available treatment options.

## 1. Introduction

Most newly diagnosed lung cancer patients develop distant metastasis, which leads to poor survival, keeping lung cancer as the leading cause of cancer-related deaths worldwide. Histological classification of lung cancer provides crucial information regarding treatment approach. Small cell lung cancer (SCLC) and non-small-cell lung cancer (NSCLC) account for the 15% and 85% of new lung cancer cases, respectively. NSCLC is further divided in three main subtypes, lung adenocarcinoma (LUAD), squamous cell lung carcinoma (SQLC) and large cell carcinoma [1]. Although a great deal of progress has been made in terms of precision medicine, particularly in oncogene-addicted disease, chemotherapy still represents a crucial treatment option for the majority of lung cancer patients, albeit with usually a short-term survival benefit. The development of tyrosine kinase inhibitors (TKIs) and immunotherapy were major breakthroughs in lung cancer treatment providing an important survival benefit for subgroups of NSCLC. Nevertheless, resistance eventually emerges also in those single molecular alteration-dependent tumors, consequently decreasing the initial benefit [1,2]. Research strategies utilizing whole genome sequencing, increasingly applied nowadays, have led to the definition of the genomic landscape of numerous cancers. PI3K/mTOR/Akt is one of the most commonly genetically altered and deregulated oncogenic signaling pathways in cancer, including lung cancer and especially NSCLC. At the end of the 1990s, phosphatase and tensin homolog deleted in chromosome 10 (PTEN) was discovered as a tumor suppressor gene and thereafter it was demonstrated that it acts as a master negative regulator of the PI3K/mTOR/Akt pathway. Nevertheless, to date clinical trials with numerous agents targeting this pathway failed to demonstrate a solid clinical benefit.

## 2. PTEN Biology

*PTEN* is one of the most frequently inactivated tumor suppressor genes in cancer and its expression is dramatically decreased in numerous cancer types [3]. *PTEN* was originally identified in 1997, when independent groups studying frequent mutations at the 10q23 locus in chromosome 10, suggested *PTEN* as a candidate tumor suppressor gene [4]. The *PTEN* gene is structured by nine exons and encodes for a 403-aminoacid protein with dual lipid and protein phosphatase utility. While other tumor suppressor genes follow the stepwise theory (such as the hereditary or sporadic retinoblastoma), *PTEN* seems to escape from this classical, two hit model and, as was demonstrated in vivo, even a subtle decrease of its levels could increase cancer susceptibility indicating a continuum for tumor suppressor behavior [5,6].

**Figure 1:**
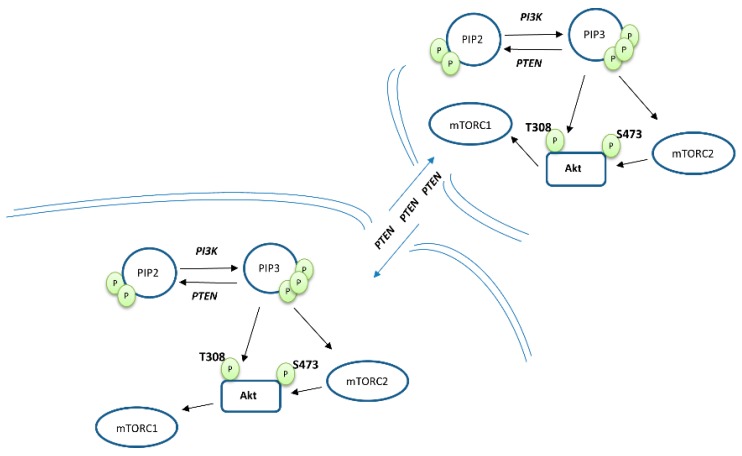
Phosphatase and tensin homolog deleted in chromosome 10 (PTEN) can exit the cell and with a paracrine manner can be engulfed by the recipient cell, exerting also there its tumor suppressor role. PI3K, phosphoinositide 3-kinase; PIP2, phosphatidylinositol 4,5-bisphosphate; PIP3, phosphatidylinositol 3,4,5-trisphosphate; mTORC1, mTOR complex 1; mTORC2, mTOR complex 2.

The PI3K/mTOR/Akt pathway is a critical oncogenic signaling that controls a plethora of cellular events. The binding of different ligands (growth factors) on receptor tyrosine kinases (RTKs) activate the PI3K either directly or through the assistance of the insulin receptor substrate (IRS) adaptor protein [7]. Then, the active PI3K phosphorylates the phosphatidylinositol-4,5-bisphosphate (PIP2) generating phosphatidylinositol-3,4,5-trisphosphate (PIP3), which acts as a lipid second messenger stimulating the activation signals to Akt and mTOR complexes initiating cell proliferation, survival and anabolic events [8]. Physiologically, PTEN patrols in proximity to this phosphorylation event preserving homeostasis by removing a phosphate group from PIP3 and converting it back to the PIP2 inactive state. In addition to this critical duty, other important findings showed that PTEN facilitates fundamental anti-oncogenic tasks, such as maintenance of chromosomal stability and competence of DNA repair by positively regulating the DNA repair protein RAD51 [9]. PTEN was described as forming homodimers for acquiring the functional lipid phosphatase configuration. However, PTEN mutants lack the catalytical function associated with wild-type (WT) PTEN units and establish, in a dominant negative manner, an inactive state. This suggests that mechanisms interfering with the dimerization could prevent its proper activity and trigger oncogenesis [10]. Phosphorylation of PTEN C-tail induces folding and association with the rest of the protein body, promoting a closed shape of PTEN shelling the binding sites and thus negatively regulating its tumor suppressor function. Nevertheless, eradication of the phosphorylation residues could restore PTEN to fully active conformation [11]. Interestingly, both PTEN and PTEN-L, an alternative less abundant transcriptional variant, 173 amino acids longer, were found to be secreted in the extracellular environment exerting their tumor suppressor properties into the recipient cells in a cell autonomous manner, a finding that might imply pharmaceutical utility for PTEN delivery (Figure 1) [12,13,14]. It is noteworthy that with the presence of two different PTEN peptides, two homodimers (PTEN/PTEN, PTEN-L/PTEN-L) and one heterodimer (PTEN/PTEN-L) can be formed, likely enriching the spectrum of mediated activities [15].

**Figure 2:**
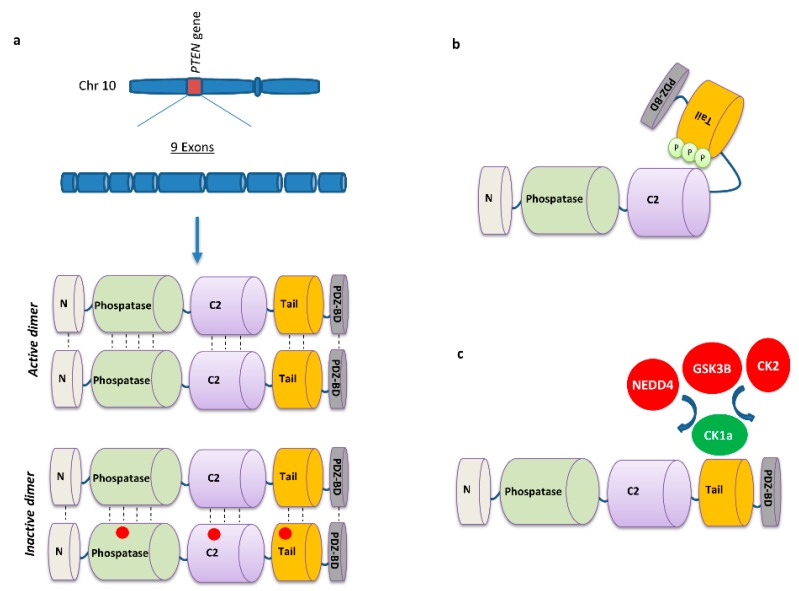
Multidisciplinary regulatory mechanisms of PTEN expression and function. (**a**) *PTEN* is located on human chromosome 10q23.3 and contains nine exons, encoding for a multidomain protein. PTEN activity can be enhanced by its homodimerization, whereas mutated PTEN peptides render the homodimer inactive. (**b**) Phosphorylation of the PTEN C-terminal tail induces an interaction with the C2 domain, promoting the folding of the tail establishing a closed and inactive conformation. (**c**) PTEN is a phosphorylation and ubiquitination target for different factors which lead to inactivation/degradation (CK2/NEDD4), while other factors exert a protective role (CK1a). CK2, casein kinase 2; GSK3B, glycogen synthase kinase 3 beta; NEDD4, neural precursor cell-expressed developmentally downregulated protein 4; CK1α, casein kinase 1 alpha 1.

**Figure 3:**
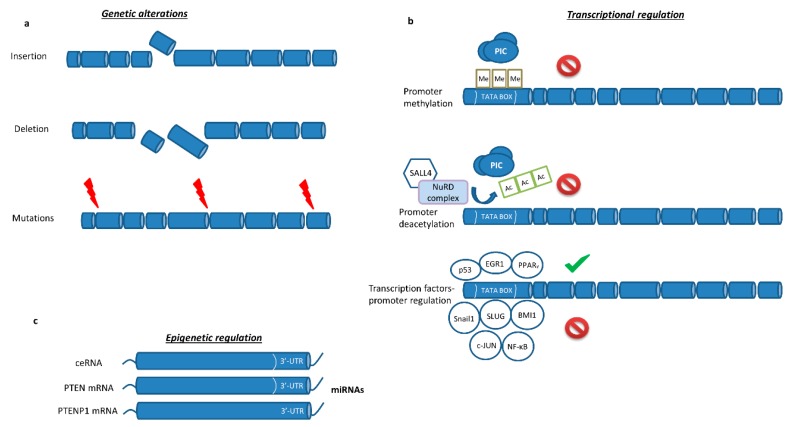
Additional multidisciplinary regulatory mechanisms of PTEN expression and function. (**a**) PTEN regulation by genetic mechanisms involves insertion, deletion and mutations that lead to functionally impaired PTEN peptides. (**b**) At a transcriptional level, *PTEN* promoter can be found heavily methylated or deacetylated, abrogating transcription initiation. Moreover, different transcription factors bind to promoter region and either promote transcription (PPARγ, etc.) or repress it (BMI1, NF-κB, etc.). (**c**) The 3′-UTR of PTEN mRNA is a direct target of numerous miRNAs, which negatively regulate PTEN expression in a post-transcriptional level. However, *PTEN* pseudogene transcript as well as other protein-coding mRNA transcripts function as decoy, ameliorating PTEN downregulation. PIC, preinitiation complex; SALL4, SAL-like protein 4; NuRD complex, nucleosome remodeling and deacetylase complex; PPARγ, peroxisome proliferator-activated receptor gamma; BMI1, polycomb complex protein BMI1; NF-κB, nuclear factor kappa beta.

PI3K/mTOR/Akt is usually aberrantly activated due to gene amplification and the gaining of function mutations of the catalytic subunits of PI3K or loss of function of the tumor suppressor gene *PTEN* [16]. According to the Cancer Genome Atlas (TCGA) research network, *PTEN* genetic alterations in SQLC are found in approximately 15% of cases, while they are much less frequent in LUAD (3%). These rates cannot explain the high prevalence of PTEN protein loss usually observed in lung cancer samples [17,18]. Therefore, other modification mechanisms should be involved. Indeed, *PTEN* gene expression is tightly regulated by genetic, epigenetic-driven mechanisms, as well as through protein–protein interactions (Figure 2 and Figure 3).

### 2.1. PTEN Epigenetic Regulation

At the epigenetic level, SAL-like protein 4 (SALL4) recruits a complex with nucleosome remodeling and histone deacetylate activity (NuRD) at the *PTEN* locus repressing its transcription [19]. Epigenetic silencing of *PTEN* gene expression in different cancers is also mediated by aberrant hypermethylation of CpG islands on its promoter [20,21]. In early-stage NSCLC evaluated by immunohistochemistry (IHC), 24% (30/125) of samples were PTEN negative. Then, 20 PTEN negative and 10 PTEN positive samples were subjected to a methylation specific polymerase chain reaction (PCR) assay and *PTEN* promoter was methylated in 35% (7/20) of PTEN negative cases, whereas no methylation was detected in PTEN positive samples [22]. Additionally, 69% (11/16) of NSCLC cell lines harbored methylated *PTEN* promoter. The negative impact of methylation on PTEN expression was confirmed by inducing PTEN expression in a highly methylated NSCLC cell line upon the use of a demethylating agent [22]. Methylation-specific PCR assay analysis of the *PTEN* promoter in a different cohort of NSCLC samples detected methylation events in 26% (39/151), albeit methylation status is not predictor for PTEN expression since unmethylated samples lacked PTEN expression as well [23].

### 2.2. PTEN Post-Transcriptional Modulation by Non-Coding RNAs

PTEN mRNA is highly susceptible to post-transcriptional control by different non-coding RNAs in numerous cancer types including NSCLC (Table 1). Indeed, miRNAs directly targeting PTEN include miR-524 in osteosarcoma, miR-146b in thyroid cancer and miR-205 in NSCLC [24,25,26,27]. Recently, lncRNA growth arrest-specific 5 (GAS5) was reported to recognize and target miR-205, decreasing its levels and allowing PTEN to increase in NSCLC cell lines [28]. The miR-21 is frequently upregulated in numerous cancer types and has been reported to directly target and repress PTEN expression in NSCLC [29]. Furthermore, miR-449a has been detected downregulated in gefitinib resistant in vitro and in vivo NSCLC models [30]. Furthermore, higher expression levels of miR-328 and lower expression levels of PTEN were detected in cisplatin resistant compared with cisplatin sensitive NSCLC patients [31]. Another oncomiRNA manipulating PTEN levels is miR-130b, which was reported as increased in NSCLC cisplatin resistant cells. Inhibiting miR-130b overcomes cisplatin resistance both in vitro and in vivo. Its function is based on PTEN downregulation with a subsequent promotion of the Wnt/β-catenin signaling axis [32]. Indeed, PTEN has been described as a negative regulator of Wnt/β-catenin in NSCLC and prostate cancer, mainly by affecting β-catenin function [33,34]. Higher levels of miR-93-5p were found in NSCLC compared to normal tissue and patients with high expression had more dismal survival rates than those with lower expression [35]. More examples of epigenetic regulation of PTEN involve miR-29b, miR-92a, miR-183-5p, miR-374b, miR-494 and miR-4286 [36,37,38,39,40,41]. Interestingly, the transcripts of *PTEN* pseudogene 1 (PTENP1) which share highly homologous regions with *PTEN* have started to attract increasing research attention. Due to the almost identical sequence, PTENP1 transcripts act as a bait for miRNAs sacrificing themselves for allowing PTEN mRNA to escape miRNA surveillance [42]. This decoy mechanism expands also to other protein-coding mRNAs, so called competitive endogenous RNAs (ceRNAs). These ceRNAs act as targets for PTEN-targeting miRNAs, which might drive PTEN upregulation [43]. For instance, ZEB2 protein is a well-known initiator of epithelial–mesenchymal transition (EMT). However, ZEB2 mRNA acts as a ceRNA of PTEN in melanoma cells, exerting a tumor suppressor role [44].

### 2.3. PTEN Post-Translational Regulation

Protein phosphorylation is a critical post-translational modification resulting in either activation or inhibition of downstream effectors. Induced expression of a modified version of PTEN C-tail in LUAD cell line H-1299 resulted in a striking reduction of cell proliferation as compared to transduction with a WT version of PTEN. Moreover, upon leptin treatment which mediates phosphorylation of PTEN and triggers the PI3K/mTOR pathway, cells expressing the mutated PTEN form, which has compromised phosphorylation sites, proliferated with a slower rate than those with WT PTEN, likely due to the inability of leptin to phosphorylate PTEN. This finding highlights the negative regulatory role that phosphorylation events exert on the tumor suppressive function of PTEN. Interestingly, phosphorylated levels of PTEN were higher in tumor tissue and were increasing with tumor grade. The mutated PTEN binds to transcription factor E2F1, blocking the transcription of cell cycle genes (cyclin D1, cyclin E1) [45]. Although PTEN creates an antioncogenic shield above different factors, there are also guardians of PTEN protecting its function and stability. CK1α is a PTEN interacting-protein, which binds to the C-terminal tail with higher affinity than any other kinases (GSK3B, CK2) therefore preventing its phosphorylation. Moreover, the interaction of CK1α and PTEN antagonized NEDD4 binding, thus abrogating the NEDD4-mediated PTEN polyubiquitination. Interestingly, NSCLC patients with high CK1α expression had a significantly longer survival than those with low expression and this was observed only in PTEN-detectable group confirming the close association of PTEN with CK1α [46]. Another guardian of the PTEN protein is Importin-11 (IPO-11), which mediates and is required for PTEN nuclear translocation. Genetic perturbation of IPO-11 resulted in increased polyubiquitination and degradation of PTEN in cytoplasm due to its insufficient import to nucleus. Moreover, IPO-11 absence or malfunction allows NEDD4 ubiquitin ligase to approach and degrade PTEN. Similarly, in vivo findings, confirm that PTEN levels are decreased in the presence of malfunctional IPO-11. In analysis of human lung cancer samples, a strong correlation between absent or low IPO-11 with absent or low PTEN was reported [47].

### 2.4. PTEN-Transcription Factors Interaction

Transcription factor binding sites on *PTEN* promoter are direct targets of p53, early growth response protein 1 (EGR1) and peroxisome proliferator-activated receptor γ (PPARγ), which trigger transcription initiation [48,49]. On the other hand, transcription repressors Snail1 and SLUG antagonize p53 for the *PTEN*-promoter binding sites [50,51]. Moreover, *PTEN* transcription is also directly abrogated by BMI1, c-Jun and nuclear factor kappa B (NFκB) [52,53,54]. EYA2 was reported upregulated and inversely correlated with PTEN expression in lung cancer tissue and NSCLC cell lines, leading to increased lung cancer cell proliferation. Deeper experiments revealed that EYA2 upregulates the expression levels of miR-93, which in turn targets directly the 3′ UTR of PTEN downregulating its expression [55]. Other regulatory mechanisms describe Myc-driven WW domain–containing ubiquitin E3 ligase 1 (WWP1) upregulation, which in turn promotes polyubiquitination and inactivation of PTEN in prostate cancer cells. Interestingly, pharmacological inhibition of WWP1 by a natural compound derived from cruciferous vegetables can restore PTEN expression [56]. After PTEN loss in prostate epithelium, TGF-β signaling emerges as a defense barrier abrogating prostate tumorigenesis. However, the presence of chicken ovalbumin upstream promoter transcription factor II (COUP-TFII) abolishes this protective mechanism by interacting with SMAD4, a crucial downstream effector of TGF-β signaling pathway, inhibiting the pathway flux [57]. These findings should trigger lung cancer-related studies exploring novel means of PTEN regulation and potential therapeutic intervention.

## 3. PTEN Dysregulation Initiates Oncogenesis

In numerous major cancers, including NSCLC and breast cancer, PTEN limited expression or complete absence is correlated with clinical outcome and therapy efficacy [58,59,60]. The pattern of *PTEN* genomic alterations is highly diverse across cancer types and some genetic alterations could exert a stronger oncogenic effect than others (missense mutations versus truncated mutations) [61]. Regarding lung cancer oncogenesis, PTEN seems to play a role in regulation of the apical junctional complexes (polarized epithelial cells). Smoking can induce downregulation of PTEN expression (likely due to an immune-mediated mechanism) and accordingly increase mTOR/Akt signaling activation in airway epithelium of healthy and chronic obstructive pulmonary disease (COPD) smokers compared to non-smokers [62,63]. PTEN loss in epithelium of in vivo models resulted in leptin signaling initiation followed by epithelial hyperplasia. In the presence of leptin, lung adenocarcinoma *PTEN*-null cells proliferated and migrated with a higher rate through the upregulation of the oncogenic pathways PI3K/mTOR/Akt, MAPK, JAK/STAT3, establishing a positive feedback among them that was abrogated after pathway targeted inhibition [64]. KRAS-driven cancer progressed faster, in the sensitizer background of *PTEN* null. In the absence of this strong anti-oncogenic force, infiltration of neutrophils and endothelial cells was increased leading to elevated vascularization and inflammation, altering the status of the tumor microenvironment and leading to higher cell proliferation rates [65]. Intriguingly, delivery of urocanic acid-modified chitosan-mediated *PTEN* gene aerosol into lung adenocarcinoma KRAS mutant in vivo models increased PTEN levels and downregulated the oncogenic mTOR/Akt axis [66].

## 4. PTEN as an Inhibitory Factor for Metastasis

Interesting findings highlight the inhibitory role of PTEN in metastasis initiation. Genetic silencing of *PTEN* in vitro and in vivo upregulated the EMT markers (N-cadherin, vimentin), together with the induction of EMT-associated morphological modification in *PTEN*-deficient cells. Moreover, β-catenin was detected exclusively in the nucleus of *PTEN*-deficient cells in a ready-to-act state [33]. In lung cancer cells, TGF-β, a well-known promoter of EMT, increased the phosphorylation levels of PTEN while decreasing PTEN expression. Substitution of the four-Ala on the phosphorylation region in the C-terminal domain of PTEN (PTEN4A) abrogated more efficiently TFG-β mediated EMT initiation, blocking β-catenin at the cell membrane, repressing cell motility in vitro and tumor growth in vivo than WT PTEN [67]. Moreover, analysis of PTEN protein levels in a set of NSCLC in vitro models showed that H1299, a cell line with EMT phenotype, expressed the lowest PTEN levels. It is known that a hypoxia condition could ignite EMT. In NSCLC cells maintained in hypoxia conditions, PTEN levels were downregulated, EMT markers were increased and β-catenin was translocated in cytoplasm and nucleus. Induction of PTEN4A in hypoxia-cultured cells partially reversed EMT by inhibiting the translocation of β-catenin into the cytoplasm and nucleus [68].

## 5. PTEN Status and Clinical Implications in Lung Cancer

Nowadays, research strategies including whole genome screening of different cancer types are considered as the most comprehensive approach, potentially enriching the currently available therapeutic opportunities. PTEN genetic, epigenetic and expression profiles in lung cancer and the available correlation with clinicopathological factors are reported in Table 2.

### 5.1. PTEN Genetic Status in Lung Cancer

Results from different studies are consistent regarding the low rate of *PTEN* genetic alterations (2–7% approximately) [69,70,71,72]. Interestingly, PCR-based assay and direct sequencing in 176 NSCLC tissues recognized 4.5% (8/176) *PTEN* mutations on exons 5–8, with four of them described only in lung cancer. Patients with these mutations were all smokers and mostly SQLC. Assessment of the impact of one of these mutations on protein functional level reported a truncated protein of high instability [69]. Moreover, next-generation sequencing (NGS) analysis in 162 advanced NSCLC Korean patients identified four patients (2.5%, 4/162) with the following mutations: K66E, R130X, Q171X, P246L [70]. *EGFR*-mutant NSCLC tumors concurrently harbor *PTEN* mutations in 6.6% (1/15), agreeing with TCGA where *PTEN* deletion or mutation was found in 5.5% (1/18) of EGFR-exon 19 deletion or L858R mutant tumors [84]. Owing to limited tissue availability regarding SCLC, blood-based real time monitoring tools have started to receive increasing attention. Whole genome sequencing in plasma cell free DNA of 24 SCLC patients detected *PTEN* deletion in 29% (7/24), while blood samples from 99 SCLC patients subjected to targeted mutational analysis on codons 5 (p.R130G), 6 (p.R173C) and 8 (p.T319fs*1) of *PTEN*, resulted negative for each of the specified [81,82]. In contrast, SCLC plasma cell free DNA was analyzed by NGS using a panel targeting all *PTEN* exons. Insertion-deletion mutations in 7.4% (2/27) were identified, highlighting the value of cutting-edge technology and the importance of the analytic method [83].

### 5.2. PTEN Protein Status in Lung Cancer

In contrast with the low frequency of genetic alterations, the prevalence of PTEN protein loss is much higher. Most of the relevant studies have described PTEN protein loss in more than 40% of NSCLC cases while some of them suggested correlation with smoking status, SQLC histology and decreased survival [23,73,74,75,76,77,78,79,80]. For example, IHC analysis of PTEN in 289 NSCLC patients and 76 healthy or benign cases reported 59.86% (173/289) and 3.94% (3/76) to be PTEN negative, respectively. PTEN negative cases had lymph node metastasis, were usually smokers, and carried the worst survival [74]. A different cohort with 288 resected NSCLC samples reported PTEN loss in 42.4% (122/288), significantly correlated with SQLC histology, smoking status, advanced disease and larger tumor size, as well as shorter progression-free survival (PFS) than the PTEN positive cases [75]. In another study, while decreased expression of PTEN was observed similarly in SQLC and LUAD, total loss of PTEN was more common in SQLC, 21% (9/43) than LUAD 4% (2/56). Also, *PTEN* loss was associated with SQLC. In vitro NSCLC models harboring *PTEN* loss were sensitive to PI3K inhibition [77]. PTEN expression was reported totally absent in 41.4% (63/152) by IHC and was more frequent in SQLC cases. However, PTEN loss was correlated with shorter disease-free survival (DFS) only in LUAD patients. From the above cases, only the 5.6% (7/124) showed *PTEN* deletion and all were PTEN negative by IHC. Interestingly, PTEN loss in never smokers was detected only in 26%, but without significant difference from smokers [78]. High prevalence of PTEN expression loss was demonstrated also in a cohort of 102 NSCLC cases. PTEN negative by IHC was 46.1% (47/102) and 0% (0/20) of NSCLC samples and healthy tissues, respectively. The findings also suggested a negative correlation between p-Akt^S473^ and PTEN and poor survival for p-Akt^S473^ positive/PTEN negative [80]. IHC analysis of more than 1000 patients in two cohorts of predominantly early stage NSCLC cases (LUAD and SQLC), performed in independent laboratories, confirmed that cytoplasmic PI3Kβ overexpression and PTEN low or negative expression were significantly more prevalent in SQLC than LUAD. Moreover, the SQLC subtype was found to be more frequently correlated with simultaneous PI3Kβ overexpression and inverse expression of PTEN than LUAD patients. This finding suggests the potentially increased benefit by using a selective PI3K isoform inhibitor especially for SQLC that completely lacks targeted therapeutic options [85]. In 2016, a meta-analysis by Xiao et al. involving 23 studies (some of them were discussed in this review) with around 2.500 NSCLC patients concluded that decreased PTEN expression detected by IHC was associated with shorter overall survival (OS), DFS and PFS [86].

### 5.3. PI3K/mTOR/Akt Inhibition in Lung Cancer

Considering the crucial role of PI3K/mTOR/Akt signaling in cancer initiation, progression and metastatization, some inhibitors of this pathway have been tested and afterwards included in the therapeutic strategy of various cancer types. Among the approved drugs in oncology are two rapamycin analogs inhibiting the mTOR complex 1 (mTORC1): (i) temsirolimus for the treatment of advanced renal cell carcinoma and (ii) everolimus for advanced renal cell carcinoma, pancreatic neuroendocrine tumors and advanced hormone receptor-positive, HER2-negative breast cancer (in combination with exemestane). Recently, the Food and Drug Administration has granted approval for the PI3K inhibitor alpelisib for hormone receptor-positive, HER2-negative, PIK3CA-mutated breast cancer (in combination with fulvestrant). In lung cancer, several inhibitors of PI3K/mTOR/Akt axis have been evaluated in clinical trials as single agent and/or combination therapies demonstrating relevant adverse events without a solid clinical benefit. The main available studies, as well as the ongoing clinical trials with inhibitors of PI3K/mTOR/Akt signaling in lung cancer are summarized in Table 3.

## 6. PTEN-Mediated Resistance to Targeted Therapy

Development of EGFR TKIs (gefitinib, afatinib, erlotinib) was a breakthrough in the treatment of NSCLC patients carrying *EGFR* mutations. However, the emergence of resistance is almost inevitable in these oncogene-addicted patients, after roughly 12 months, predominantly due to the secondary mutation *EGFR* T790M. Additionally, other mechanisms which have been associated with EGFR TKIs resistance in NSCLC patients involve PTEN loss and the activation of PI3K/mTOR signaling [92]. Shortly after, another study reconfirmed the advent of EGFR TKI resistance in the absence of PTEN expression. Gefitinib-resistant NSCLC in vitro models showed a significant decrease in PTEN expression followed by upregulation of Akt phosphorylation compared to the sensitive parental cells. Reduced nuclear localization of EGR1, a transcription factor regulating *PTEN*, in resistant cells might represent a mechanism leading to the downregulation of PTEN expression. Moreover, genetic silencing of *PTEN* in cell lines conferred resistance to gefitinib, supporting the notion that PTEN expression is associated with EGFR TKI sensitivity [93]. Intriguingly, a single case report described a multiple primary lung cancer in which the adenocarcinoma of the left lobe was an *EGFR* mutant at exon 21 and *KRAS*/*PTEN* negative and thus, was treated with gefitinib. While the left lobe lesions responded to TKI, the poorly differentiated lesion of the right lobe progressed. After resection of the right lobe, the tissue was subjected to mutation analysis, which revealed a *PTEN* mutation, but no *EGFR* mutations [94]. PTEN proficient HCC827 cells with the sensitizing to-TKI-therapy *EGFR* mutation showed high sensitivity to gefitinib. Genetic deletion of *PTEN* diminished this sensitivity. Treatment of in vitro PTEN-deficient *EGFR*-mutant NSCLC models with PPARγ agonists (rosiglitazone, pioglitazone) in presence of gefitinib produced a strong synergism and induced PTEN protein expression and autophagy mitigating TKI resistance in NSCLC increasing the anticancer effect. The PPARγ-specific contribution was confirmed also after genetic and pharmaceutical inhibition where the reverse of the effect was described [95,96]. NEDD4 is a E3 ubiquitin ligase that promotes ubiquitin-mediated proteasomal degradation of PTEN and was moderately and strongly expressed in 26/103 and 56/103 in NSCLC samples, respectively, correlating significantly with PTEN low protein levels [97,98]. Moreover, it was found to be negatively regulated also in HCC827 TKI erlotinib resistant cells at mRNA and protein level. Interestingly, only concomitant NEDD4 genetic deletion and PTEN transfection in the PTEN-deficient H1640 resistant cells managed to increase significantly the resistance to erlotinib indicating that NEDD4-mediated resistance mechanism relies on PTEN presence and subsequent degradation [99]. As mentioned previously, methylation of the *PTEN* promoter could be found in EGFR TKIs resistant cell lines. Interestingly, treatment of these cell lines with demethylating agents could restore PTEN levels and consequently sensitivity to TKIs, suggesting that epigenetic modulators could be considered as treatment options [100]. Interestingly, in a study where 169 NSCLC patients harbored *EGFR*-sensitive mutations, those that had concurrent *PTEN* deletion showed a more dismal PFS and OS compared to those with intact *PTEN*. In addition, *PTEN* deletion and low PTEN protein expression are strong and independent predictors of worse PFS in EGFR-TKI treated NSCLC patients [101]. Moreover, high *PTEN* expression seems to contribute to prolonged survival in gefitinib-treated NSCLC patients, while *EGFR* mutant patients with concomitant elevated *PTEN* expression showed the longest survival compared to other subgroups [102]. The parallel architecture of the signaling pathways enables crosstalk either at a baseline or after the treatment pressure as a bypass mechanism. PTEN appears to participate in the circuit of PI3K and MAPK signaling pathways that cooperate for the interest of cell survival and proliferation. PTEN assessment in a set of lung cancer cell lines resulted in variable outcomes among them. Interestingly, a targeted combination of everolimus with trametinib (a MEK inhibitor) produced a synergistic effect in a large panel of cell lines lacking PTEN expression, including cells of other origin besides lung cancer, while PTEN competent cell lines exhibited either a slight additive or antagonistic effect. Combined inhibition of MEK and both mTOR complexes using double mTOR or PI3K/mTOR inhibitors might result in a more profound response and therefore deserves attention [103].

## 7. PTEN Role in Tumor Microenvironment and Immunotherapy Sensitivity

The tumor microenvironment frequently serves as a major limitation for current anti-cancer drugs. Nevertheless, immunotherapy has brought a strong wind of optimism in lung cancer treatment with an already reported remarkable survival benefit for NSCLC patients. The delineation of predictive biomarkers has lagged behind and if steps would be taken forwards, the expected profit from immunotherapy will be improved, reducing the proportion of patients without benefit [104]. The presence of T regulatory cells (T_regs_) is fundamental for controlling immune response thus providing protection from autoimmune disorders and appropriate regulation of PI3K/mTOR signaling is necessary for this [105]. T_regs_ specific deletion of *PTEN* in vivo disturbed the immunotolerance inducing spontaneous inflammatory conditions. PTEN is required for the stability of T_regs_ controlling transcription and metabolic events silencing mTOR complex 2 (mTORC2) axis [106]. Lewis lung carcinoma (LLC) tumors implanted in hosts with *PTEN*-KO T_regs_ grew with a slower rate than LLC tumors in WT hosts. Moreover, concomitant administration of chemotherapy and VO-OHpic (a PTEN inhibitor) exerted a synergistic effect reducing LLC tumor size. This drug combination promotes a proinflammatory phenotype within a tumor microenvironment that was quite similar with that observed in *PTEN*-KO T_regs_ hosts. Although this approach can raise concerns for PTEN expressing patients, it might harbor therapeutic value for PTEN-deficient patients [107]. From a technical perspective, PTEN basal expression in stromal cells can be utilized as a control marker of tissue quality and, according to the acceptable expression levels, tissues can be analyzed with for PD-L1 or rejected. While exclusion of poor-quality samples is critical before biomarker analysis, the criteria for selecting PTEN as a tissue quality marker should be explored deeper [108]. IFN-γ-mediated anti-oncogenic functions are well demonstrated in cancer, including in lung cancer, and exogenous administration has been used to treat different malignancies and PTEN appears to play a role also in the IFN-γ impact. While PTEN competent A549 cell respond to IFN-γ, genetic deletion of *PTEN* in them but also PTEN-deficient PC14PE6/AS2 cells failed to respond. Moreover, after exploring the negative regulators of IFN-γ/JAK2/STAT1 axis, generation of ROS was reported which in turn increased the p-SHP2, a phosphatase that can deactivate JAK2/STAT1. Treatment of the cells with antioxidant N-acetyl-cysteine or SHP2 inhibitor decreased the SHP2 levels and restored the IFN-γ mediated antioncogenic effect [109]. PTEN hamartoma tumor syndrome (PHTS) individuals harbor germline *PTEN* mutations and characterized by an increased predisposition for benign and malignant tumorigenesis. As expected, inactive PTEN results in a higher activity of PI3K/mTOR/Akt pathway in PHTS patients leading to increased aerobic glycolysis generating lactate, a modulator of the immune system, enhancing a proinflammatory environment [110]. In the pursuit of identifying reliable predictive biomarkers, glioblastoma patients who did not respond to anti-PD-1 therapy were analyzed and reported to be significantly enriched in *PTEN* mutations, especially loss-of-function mutations within the C2 domain, leading to the establishment of a immunosuppressive context and an upregulated PI3K/mTOR pathway activity as was showed in non-responders patients with *PTEN* mutations [111]. Immunotherapy resistance mediated by PTEN loss is also confirmed in metastatic uterine leiomyosarcoma and melanoma [112,113]. Interestingly, immunosuppressive cytokines such as VEGF have been reported overexpressed in PTEN negative tumors. Clinical trials including anti-PD-1 combined with anti-vascularization agents are currently ongoing including NSCLC (NCT02443324). A phase III study showed the combination of the anti-PD-L1 avelumab with the multi-tyrosine kinase inhibitor axitinib is superior to sunitinib and led to a longer PFS in advanced renal-cell cancer patients [114]. Interestingly, a metastatic NSCLC case with *PTEN* mutation, 80% PD-L1 expression and high tumor mutational load showed a durable response to temsirolimus (mTORC1 inhibitor), whereas treatment with anti-PD-1 antibodies induced rapid progression. These findings demand further investigation for exploring the potential superior biological impact of *PTEN* mutations over immunotherapy sensitizing mechanisms and/or the PTEN-mediated resistance to immunotherapy despite candidate immunologic predictive markers [115].

## 8. Conclusions

PTEN is a powerful tumor suppressor gene that counteracts several oncogenic stimuli and it has been demonstrated that even partial PTEN protein loss could initiate carcinogenesis. While the most critical duty of PTEN is the negative regulation of the PI3K/mTOR/Akt oncogenic pathway, thus inhibiting uncontrolled cell survival, growth and migration, further crucial antioncogenic functions have been attributed to PTEN. Intriguingly, large cohort studies analyzing human lung cancer samples showed decreased and complete loss of PTEN expression for 40% of cases, approximately. However, genetic alterations of *PTEN* (mutations/deletion) in lung cancer do not match the protein loss prevalence and, therefore, non-genomic mechanisms regulating PTEN expression should exist. Indeed, epigenetic (miRNAs, methylation, acetylation) and post-translational (phosphorylation, oxidation) mechanisms have been preclinically and clinically detected. Since intervention on a genetical level is still an unmet need, epigenetic pharmaceutical modulation by demethylating agents and anti-miRNA oligonucleotides represents an attractive challenge. Potential combinations involving perturbation of PTEN loss-driven signaling pathways through targeted inhibitors (PI3K/mTOR/Akt) might produce a more profound anti-oncogenic effect. Efforts to modulate PTEN levels were reinforced further by the findings which associate *PTEN* mutations with resistance to TKI and recently to immunotherapy. Given the intricate multilevel regulation of PTEN, a global assessment of PTEN status in a retrospective and perspective manner could identify initially harboring or post treatment arising genetic/protein alterations leading to the identification of a PTEN signature with predictive and/or prognostic value. PTEN protein levels have been evaluated by IHC in numerous studies within large cohorts and valuable information regarding histology and prognosis was extracted. For the time being, PTEN is evaluated in clinic by IHC, an indispensable diagnostic tool and the most appropriate and cost-effective method for estimating PTEN protein expression on lung cancer tissue. However, pathologist visual scoring, specificity of the antibody and tissue quality are some of the main IHC limitations. Evaluation by independent pathologists, proper handling and preparation of the tissue but also PTEN antibody validation in *PTEN*-null genetic models could ameliorate potential bias. Until recent years, PTEN was not considered according to its high tumor suppressor value by the research community, while at the same time the downstream signaling network, and especially the PI3K/mTOR/Akt pathway, received enormous attention. However, first rate discoveries regarding PTEN structure and the mechanisms controlling its activation, together with the advent of pioneer technology, will accelerate the elucidation of PTEN function and regulation, potentially leading to more clinically meaningful therapeutic approaches for lung cancer patients.

## Figures and Tables

**Table 1:** Role of different PTEN-targeting miRNAs in non-small-cell lung cancer (NSCLC) prognosis and therapy resistance.

microRNA	Expression	Material	Clinical Correlation	Ref.
miR-21	High	Tissue/in vitro	Chemo- and radio resistance	[29]
miR-29b	Low	Tissue/in vitro	LN metastasis	[36]
miR-92a	High	Tissue	Tumor stage/LN metastasis	[37]
miR-93-5p	High	Tissue	Poor survival	[35]
miR-130b	High	In vitro/In vivo	Cisplatin resistance	[32]
miR-183-5p	High	Tissue/in vitro	Tumor volume	[38]
miR-205	High	Tissue/in vitro	Chemo resistance	[26,27,28]
miR-328	High	Tissue/in vitro	Cisplatin resistance	[31]
miR-374b	High	Tissue/in vitro	NR	[39]
miR-449a	Low	EGFR TKI res NSCLC tissue/in vitro/in vivo	EGFR TKI resistance	[30]
miR-494	High	Tissue	LN metastasis/poor OS	[40]
miR-4286	High	Tissue/in vitro	Histology	[41]

Ref., reference; NR, not reported; OS, overall survival; NSCLC, non-small cell lung cancer; LN, lymph nodes; Gef res, gefitinib resistant models.

**Table 2:** PTEN genetic, epigenetic and expression profile in lung cancer and correlation with clinicopathological factors.

Histologic Type	Finding	Number of Patients	Human Material	Correlated Parameters	Discovery Technique	Ref.
NSCLC	Protein loss, Promoter methylation	24% (30/125), 35% (7/20)	Tissue	NR	IHC, PCR	[22]
NSCLC	Promoter methylation	26% (39/151)	Tissue	No predictor of protein expression	PCR	[23]
NSCLC	Mutation	4.5% (8/176)	Tissue	smokers, mostly SQLC	PCR, sequencing assays	[69]
A-NSCLC	Mutation	2.5% (4/162)	Tissue	NR	NGS	[70]
LUAD	Mutation	2.2% (1/45)	Tissue	NR	NGS	[71]
LUAD	Deletion	6.8% (2/29)	Tissue	NR	NGS	[72]
NSCLC	Protein loss, mutation	50% (86/173), 4% (7/180)	Tissue	NR	IHC, PCR	[73]
NSCLC	Protein loss, weak	44% (52/117), 29% (34/117)	Tissue	Stage I and II	IHC	[22]
NSCLC	Protein loss	59.86% (173/289)	Tissue	LN metastasis Smoking status, Decreased survival	IHC	[74]
NSCLC	Protein loss	42.4% (122/288)	Tissue	SQLC, Smoking status; Decreased PFS	IHC	[75]
NSCLC	Protein loss	39% (41/104)	Tissue	More prevalent in SQLC	IHC	[76]
SQLC, LUAD	Protein loss	21% (9/43), 4% (2/56)	Tissue	PTEN gene loss was associated with SQLC	IHC	[77]
NSCLC	Protein loss, Deletion	41.4% (63/152), 5.6% (7/124)	Tissue	More prevalent in SQLC, shorter DFS for LUAD	IHC, FISH	[78]
NSCLC	Protein loss	41.4% (43/104)	Tissue	Advanced disease, LN metastasis, Decreased survival	IHC	[79]
NSCLC	Protein loss	46.1% (47/102)	Tissue	Poor survival for p-Akt^S473^ positive and PTEN negative	IHC	[80]
SCLC	Deletion	29% (7/24)	cf-DNA	NR	WGS	[81]
SCLC	Mutation	0% (0/99)	cf-DNA	NR	HRM	[82]
SCLC	Mutation	7.4% (2/27)	cf-DNA	NR	NGS	[83]

Ref., reference; NR, not reported; IHC, immunohistochemistry; PCR, polymerase chain reaction; NGS, next-generation sequencing; WGS, whole genome sequencing; FISH, fluorescent in situ hybridization; HRM, high resolution melt; OS, overall survival; RFS, relapse free survival; A-NSCLC, advanced-non-small cell lung cancer; LUAD, lung adenocarcinoma; SQLC, squamous cell lung cancer; SCLC, small-cell lung cancer; LN, lymph nodes; cf-DNA, cell-free DNA; PFS, progression-free survival; DFS, disease-free survival.

**Table 3:** Main clinical trials with PI3K/mTOR/Akt inhibitors in lung cancer.

Agent	Target	Phase	Setting	Main Results	Ref.
Everolimus	mTORC1	II	Pretreated NSCLC	DCR 47.1%; mPFS 2.6–2.7 months	[87]
Everolimus (+ erl vs. erl)	mTORC1	IIR	Pretreated NSCLC	mPFS 2.9 vs. 2.0 months; G3/4 AEs 72.7% vs. 32.3%	[88]
Everolimus (+ docetaxel)	mTORC1	II	Pretreated NSCLC	6-month PFS 5%, mOS 9.6 months	[89]
Everolimus (+ thoracic RT)	mTORC1	I	Locally advanced or metastatic untreated NSCLC	Reccommended dose with RT 50mg/week; relevant pulmonary AEs	[90]
Temsirolimus	mTORC1	II	Advanced NSCLC	mPFS 23 months, mOS 6.6 months	[91]
Vistusertib	mTORC1/2	II	Advanced RICTOR-amplified SCLC	Ongoing	NCT03106155
MK-2206	Pan-AKT	II	PI3KCA, AKT and PTEN-mutant NSCLC and SCLC	Ongoing	NCT01306045
Gedatolisib (+ carbo-paclitaxel)	Dual PI3K and mTORC1/2	I/II	Pretreated NSCLC	Ongoing	NCT02920450
Gedatolisib (+ palbociclib)	Dual PI3K and mTORC1/2	I	Squamous pretreated NSCLC	Ongoing	NCT03065062

R, randomized; ref., reference; erl, erlotinib; NSCLC, non-small cell lung cancer; mPFS, median progression-free survival; RT, radiotherapy; AEs, adverse events; mOS, median overall survival.
